# Insights into human evolution from 60 years of research on chimpanzees at Gombe

**DOI:** 10.1017/ehs.2021.2

**Published:** 2021-01-11

**Authors:** Michael Lawrence Wilson

**Affiliations:** 1 Department of Anthropology, University of Minnesota, 395 Humphrey Center, 301 19th Ave. S., Minneapolis, MN 55455, USA; 2 Department of Ecology, Evolution and Behavior, University of Minnesota, 140 Gortner Laboratory, 1479 Gortner Avenue, Saint Paul, MN 55108, USA; 3 Institute on the Environment, University of Minnesota, 1954 Buford Avenue, Saint Paul, MN 55108, USA

**Keywords:** Chimpanzee, *Pan troglodytes*, human evolution, Gombe National Park

## Abstract

Sixty years of research on chimpanzees (*Pan troglodytes*) at Gombe National Park, Tanzania have revealed many similarities with human behaviour, including hunting, tool use and coalitionary killing. The close phylogenetic relationship between chimpanzees and humans suggests that these traits were present in the last common ancestor of *Pan* and *Homo* (LCA_PH_). However, findings emerging from studies of our other closest living relative, the bonobo (*Pan paniscus*), indicate that either bonobos are derived in these respects, or the many similarities between chimpanzees and humans evolved convergently. In either case, field studies provide opportunities to test hypotheses for how and why our lineage has followed its peculiar path through the adaptive landscape. Evidence from primate field studies suggests that the hominin path depends on our heritage as apes: inefficient quadrupeds with grasping hands, orthograde posture and digestive systems that require high-quality foods. Key steps along this path include: (a) changes in diet; (b) increased use of tools; (c) bipedal gait; (d) multilevel societies; (e) collective foraging, including a sexual division of labour and extensive food transfers; and (f) language. Here I consider some possible explanations for these transitions, with an emphasis on contributions from Gombe.

## Graphical abstract

**Figure:**
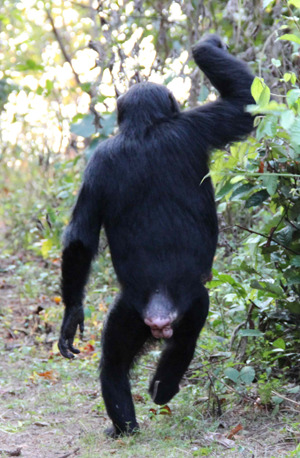

**Media summary:** Long-term study of chimpanzees at Gombe, Tanzania, begun by Jane Goodall 60 years ago, continues to shed light on key questions in human evolution.

## Introduction

In 1960, Jane Goodall established the first long-term field study of chimpanzees (*Pan troglodytes*) at what is now Gombe National Park, Tanzania, just over a century after *On the origin of species* (Darwin, 1859) laid the foundation for an evolutionary understanding of human origins. Goodall's mentor, Louis Leakey, hoped that studying living apes would shed light on the behaviour of fossil apes such as *Proconsul* (Peterson, 2006). Leakey believed the ape and human lineages had diverged deep in time (Leakey, 1970), but nonetheless thought studies of living apes would provide essential context for understanding human evolution. Fossils provide indispensable evidence, but can offer only limited information about the living creatures that left those remains behind. What sort of societies did they have? What did they eat? How did they behave?

Leakey's hopes have been rewarded abundantly. The Gombe chimpanzee study has continued for 60 years, producing over 300 scientific publications and reaching a broad global audience through popular books, magazine articles and films. Additionally, other studies that follow Gombe's model of collaborative long-term field research have broadened our understanding of living primates, providing invaluable comparative data for testing hypotheses about how and why humans evolved our many distinctive traits.

Research at Gombe revealed that chimpanzees, like humans, make and use tools (Goodall, 1964), hunt in groups and share meat (Goodall, 1963), and have hostile intergroup relations, including fatal attacks (Goodall et al., 1979). Molecular evidence of the close genetic relationship between humans and chimpanzees (Sibley & Ahlquist, 1984) suggested that chimpanzees provide a window into the lives of our common ancestors: male-dominated, warlike, monkey hunters (Ghiglieri, 2000; Goodall, 1990; Wrangham & Peterson, 1996). Others have emphasized the challenges posed to this view by our other closest living relatives, bonobos (*Pan paniscus*): female-dominated pacifists that defuse tension with sex (Parish & de Waal, 2000). Still others have argued that neither species of *Pan* is particularly similar to our common ancestor (Sayers & Lovejoy, 2008; White et al., 2012). An extreme version of this argument holds that humans are too distinct for any comparisons with *Pan* to be useful (Fuentes, 2018; Marks, 2002). Efforts to reconstruct traits of the last common ancestor of *Pan* and *Homo* (LCA_PH_) remain speculative, particularly given the sparse fossil record for African apes in the late Miocene (McNulty, 2010; Andrews, 2020). Chimpanzees and bonobos are our cousins, not our ancestors. Evolution has continued for both panins and hominins. Both living panins probably differ in important ways from our common ancestor. At the same time, the LCA_PH_ probably resembled *Pan* more than *Homo* in many ways that are important for understanding human evolution. Moreover, the many ways in which humans differ from *Pan* – and indeed, from all other species on Earth – depend on features peculiar to the apes, rather than features common to primates in general. Long-term studies at Gombe and other sites have yielded rich datasets for clarifying our place in nature. These data help explain why, among the primates, only apes produced a lineage that evolved bipedal locomotion, cumulative culture, cooking and language.

Please note that a short review such as this cannot hope to do justice to Gombe's 60 years of research, much less to the numerous studies at other sites across Africa, which have revealed considerable behavioural diversity across *Pan*. For more breadth and depth, see Arcadi (2018), Boesch et al. (2019), Furuichi (2019), Hare and Yamamoto (2015), Hunt (2020), Nakamura et al. (2015) andStanford (2018).

## Research at Gombe

Gombe National Park is located in northwestern Tanzania (latitude 4°38′–4°45′S, longitude 29°36′–29°39′E). The park covers 35.69 km^2^ of land and 20.72 km^2^ of Lake Tanganyika. Mountains rise from the lakeshore (766 m a.s.l.) to peaks (1,300–1,623 m a.s.l.) along the eastern boundary. Streams running west to the lake divide the park into a series of steep valleys. The valley bottoms contain evergreen forest, with semi-deciduous forest and vine tangle on the slopes, open woodland on the ridges and montane grassland at higher elevations. This mosaic habitat resembles habitats constructed for early hominins (e.g. Levin et al., 2008). Approximately 90 chimpanzees live in the park, divided into three communities: Mitumba, Kasekela and Kalande (Wilson et al., 2020). Other primates found in the park include olive baboons (*Papio anubis*), red colobus monkeys (*Piliocolobus tephrosceles*) and vervets (*Chlorocebus pygerythrus*), along with two species of guenon (red-tailed monkeys (*Cercopithecus ascanius schmidti*) and blue monkeys (*C. mitis doggetti*)), and their hybrids.

Goodall's books provide vivid accounts of her early years at Gombe, the subsequent development of Gombe Stream Research Centre and the chimpanzees, with their distinct personalities, rich emotional lives, complex societies and strong social bonds (Goodall, 1986, 1990; van Lawick-Goodall, 1971). While popular media often depict Goodall as a lone woman in the forest, she established and maintained an intensely collaborative research team. She founded the Jane Goodall Institute, which maintains a team of approximately 50 Tanzanian employees at Gombe to collect data on chimpanzees, baboons, guenon monkeys and plants. Research teams conduct focal follows of individual chimpanzees each day, endeavouring to follow the focal individual from dawn to dusk (Wilson, 2012). Researchers also collect observations of mothers and infants and adolescent females, monitor chimpanzee health, collect faecal and urine samples, and carry out monthly phenology transects focused on plant species important in the chimpanzee diet. Starting in the 1990s, Anne Pusey led efforts to digitize the long-term data and develop a computer database. A consortium of principal investigators at Gombe (D. Mjungu and D. A. Collins), the Jane Goodall Institute-USA (L. Pintea) and faculty at six universities (K. Detwiler, I. C. Gilby, E. V. Lonsdorf, C. M. Murray, A. E. Pusey and M. L. Wilson) now work to maintain various datasets and coordinate ongoing research.

Gombe exemplifies what has become a standard approach for primate field studies: collaborative research, collecting systematic information on identified individuals, followed throughout their entire lives. This approach was pioneered in 1948, when Kinji Imanishi and Jun'ichiro Itani began the first study of Japanese macaques (*Macaca fuscata*; Matsuzawa & McGrew, 2008). Like Leakey, Imanishi developed a broad vision of studying human evolution through combined studies of fossil remains, non-human primates and human hunter–gatherers, and in 1958 began expeditions to Africa, which eventually led to the establishment of multiple long-term studies of great apes, including chimpanzees at Mahale, Tanzania (Nishida, 2012), and bonobos at Wamba, Democratic Republic of Congo (Kano, 1992). However, Goodall was unaware of Japanese primatology when she began her research; although Itani and Imanishi were among her earliest scientific visitors at Gombe, lack of fluency in a common language prevented meaningful exchange of scientific ideas (Goodall, personal communication). Similarities in their research methods thus resulted from convergent cultural evolution, rather than vertical transmission.

Gombe serves multiple functions. As a site for observing animals going about their business in their natural habitat, it provides scientists with opportunities to learn about natural behaviour. Conducting field research inspires new scientific thinking, particularly when animals behave in unexpected ways. As Toshisada Nishida observed after decades of fieldwork at Mahale, ‘chimpanzees are always new to me’ (de Waal, 2011). As a site for systematic long-term data collection, Gombe enables scientists to test hypotheses that require more data than can easily be obtained in a single field season (or even in an entire career). As a site for training young scientists, Gombe provides opportunities for learning how to ask questions of nature in ways that (hopefully) get clear answers. At least 59 graduate students have completed doctoral dissertations based on Gombe research, and several additional dissertation projects are currently underway. Additionally, research at Gombe promotes conservation in and around the park (Wilson et al., 2020).

Studies at Gombe have covered many topics relevant for understanding human evolution, including diet (Goodall, 1963; O'Malley & Power, 2014; Wrangham, 1977), locomotion and posture (Hunt, 1994), tool use (Goodall, 1964; Musgrave et al., 2020; Whiten et al., 1999), cooperation and food sharing (Gilby, 2006; Silk, 1978; Stanford, 1996), sexual behaviour (Feldblum et al., 2014; Tutin & McGinnis, 1981; Wroblewski et al., 2009), aggression and competition (Bygott, 1979; Feldblum et al., 2018; Foerster et al. 2016; Goodall et al., 1979; Murray, 2007; Wilson et al., 2014), life histories (Alberts et al., 2013; Hill et al., 2001; Kirchhoff, 2019; Pusey, 1990; Walker et al., 2018; Williams et al., 2008) and vocal communication (Marler, 1976; Mitani et al., 1992).

The Gombe team has sought to obtain a balanced view of chimpanzee life. All-day follows of individual males and females, conducted regularly since the 1974, ensure that researchers have detailed data on both sexes and their interactions (Goodall, 1986). While accounts of predominantly male behaviours such as hunting and fighting might particularly draw public attention, much research at Gombe has focused on female social behaviour and ecology. Having studied chimpanzee feeding ecology at Gombe, Richard Wrangham placed females at the centre of theoretical models of primate social behaviour: fertile females are the key resource over which males compete, and competition among females for food shapes their spatial distribution and social relationships, and thus constrains male options (Wrangham 1979, 1980). Dominance interactions among females are more subtle than those among males, but females can nonetheless be assigned ranks (Pusey et al., 1997; Foerster et al., 2016). High-ranking females reproduce more quickly (Pusey et al., 1997; Jones et al., 2010), and maintain a more stable body mass (Pusey et al., 2005), probably because they forage in core areas with more food resources (Murray et al., 2006; Murray, 2007). They do sometimes hunt, but tend to take sedentary prey (Gilby et al., 2017). Females occasionally engage in severe aggression, particularly towards other females and their offspring (Pusey et al., 2008). Chimpanzee mothers spend years caring for their offspring; yet mothers also vary in their skill and aptitude (Goodall, 1986). Data collection focusing on mothers and infants began in 1969 and continues to the present, yielding many insights into topics including the consequences of variation of maternal behaviour (Stanton et al., 2014) and relationships among siblings (Lonsdorf et al., 2018).

## The last common ancestor of Pan and Homo

When Goodall arrived at Gombe, scientists knew little about the behaviour of apes in the wild. Gorillas and chimpanzees were assumed to be each other's closest relatives; some authorities classified them together in the genus *Pan* (Tuttle, 1969). Bonobos were known to exist but had not been studied. By the 1980s, when molecular studies began to clarify that *Pan* formed a clade with *Homo*, not *Gorilla* (Sibley & Ahlquist, 1984), field studies had documented many behavioural similarities between chimpanzees and humans. Like humans, but unlike gorillas, chimpanzees hunt, share meat, make and use tools, and live in multimale, multifemale communities with fission–fusion grouping patterns and hostile, sometimes deadly, intergroup relations (Goodall, 1986). Female chimpanzees typically leave their natal communities at sexual maturity (Nishida & Kawanaka, 1972; Pusey, 1979), similar to residence patterns reported for hunter–gatherers (Ember, 1978; although more recent analysis of hunter–gatherer residence patterns reveals a more nuanced pattern – Hill et al., 2011). Such female dispersal explained sex-biased patterns of cooperation: males cooperated with male kin, while dispersing females became socially more isolated (Wrangham, 1979). These numerous similarities seemed likely to be homologies, shared by descent from the LCA_PH_ (Ghiglieri, 1987; Wrangham, 1987), reflected in Wrangham's (2001) proposed name for the LCA_PH_: *Pan prior*, ‘the early chimpanzee’. Muller et al. (2017) explore in detail the implications of a chimpanzee-like LCA_PH_ for human evolution.

Field studies also revealed many differences. In contrast to chimpanzees, humans have bipedal locomotion; greater reliance on tools to acquire and prepare foods; more sharing of foods; home bases for cooking and sleeping; long-term breeding bonds and multilevel societies composed of multiple families; and language (Isaac, 1978).

Assuming that the LCA_PH_ was similar to a chimpanzee provides a clear starting point for efforts to explain the origin of distinctively human traits. This view has been challenged, however, on various grounds. Some consider the fossil evidence of Miocene apes and early hominins such as *Ardipithecus* to be incompatible with a chimpanzee-like LCA_PH_ (Sayers & Lovejoy, 2008; White et al., 2012). Pilbeam and Lieberman (2017) address these arguments in detail. More could be said on this topic, but here I focus on field studies of living apes.

Bonobos are just as closely related to humans as are chimpanzees, but differ in ways that complicate efforts to infer traits of the LCA_PH_ (Parish & de Waal, 2000). Bonobo field studies did not begin until 1973 (Kano, 1992), and bonobos remain less intensively studied than chimpanzees, owing to political instability and war in Congo. Nonetheless, researchers have persevered at several long-term field sites; their findings increasingly challenge preconceptions about *Pan*. Bonobos rarely use tools (Furuichi et al., 2015). They hunt infrequently, and females catch mammalian prey as often as males (Surbeck & Hohmann, 2008). As in chimpanzees, male bonobos stay in their natal groups and females disperse, but in bonobos females, rather than males, form coalitions and dominate the opposite sex (Tokuyama & Furuichi, 2016). Bonobos sometimes engage in intergroup hostility, but members of different communities can also spend days together (Sakamaki et al., 2018; Pisor & Surbeck 2019), and have been observed to share food across borders (Fruth & Hohmann, 2018). Bonobos engage in less violence than chimpanzees, and have not been confirmed to kill other bonobos (Wilson et al., 2014). In some respects, bonobos bear a closer resemblance to humans than do chimpanzees, such as in their greater elaboration and diversity of sexual behaviour, and their lower levels of reactive aggression (Parish & de Waal, 2000; Wrangham, 2019). Surprisingly, although bonobos are commonly depicted as having relaxed sexual relations, genetic studies have revealed that high-ranking males nonetheless sire a disproportionate share of offspring, with reproductive skew higher than for chimpanzees (Surbeck et al., 2017). Variation among chimpanzee populations suggests that fairly subtle changes in ecology can lead to substantial differences in social behaviour, and even physiology. Western chimpanzees appear to be more bonobo-like than eastern chimpanzees, with more cohesive parties (Furuichi, 2009; Wrangham, 1999), female sexual swellings that resume sooner after giving (Deschner & Boesch, 2007), and a tendency towards lower rates of lethal aggression (Wilson et al., 2014).

Researchers have variously argued that bonobos are more derived than chimpanzees (Pilbeam & Lieberman, 2017), less derived than chimpanzees (Diogo et al., 2017) or that both species of *Pan* are derived compared with the LCA_PH_ (Parish & de Waal, 2000). Bonobos are sometimes claimed to more closely resemble hominins, such as by having longer legs and more frequent bipedality (Zihlman et al., 1978). Controlling for body mass, however, bonobos and chimpanzees do not differ in limb length (Druelle et al., 2018), nor do bonobos engage in bipedal posture more often than chimpanzees, either in captivity (Videan & McGrew, 2001) or in the wild (Doran & Hunt, 1994). Studies of cranial development in African apes, including the chimpanzees of Gombe, challenge simple characterizations of any African ape species being either primitive or derived (Massey, 2018). Given that both species are our cousins, not our ancestors, both species probably differ in multiple ways from our common ancestor.

**Figure 1.**
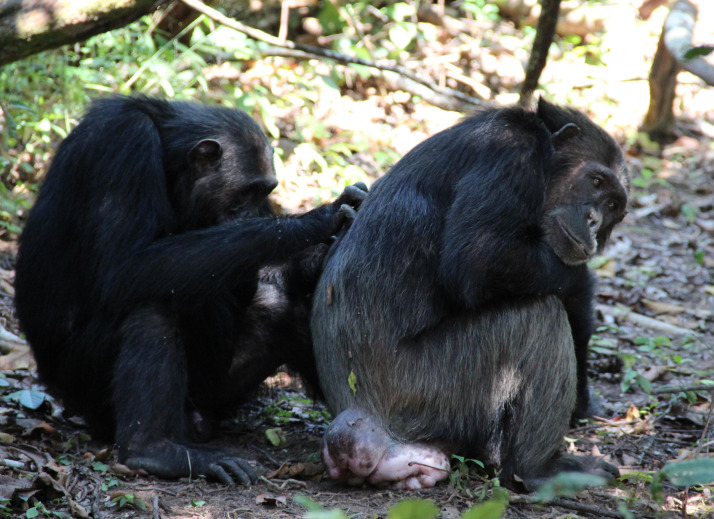
Kasekela male Faustino grooming Nasa, a female with a fully tumescent sexual swelling. Photo by Michael L. Wilson.

Using phylogenetic methods to infer likely traits of the LCA_PH_, Duda and Zrzavý (2013) conclude that both chimpanzees and bonobos are derived. For example, they conclude that prominent sexual swellings of adult females (Figure 1) are a derived trait of *Pan*. This possibility has implications for the socioecology of the LCA_PH_, since such sexual swellings occur mainly in multimale, multifemale societies (Nunn, 1999). Duda and Zrzavý (2013) reconstruct the LCA_PH_ as living in gorilla-like groups, with a single male and multiple females, and considerable sexual dimorphism. Chimpanzees are thought to be unable to live in such groups because their dependence on ripe fruit imposes severe feeding competition during fruit-poor seasons (Wrangham, 1979). Gorillas can forage together because their large size enables them to efficiently digest abundant fibrous vegetation, reducing feeding competition (Wrangham, 1979). Hunt (2016) argues that Miocene apes faced less feeding competition from monkeys, making stable groups viable for frugivorous apes. If this is the case, various other features of chimpanzee societies – fission–fusion dynamics, multimale groups, male coalitions, coalitionary killing – might not have been present in the LCA_PH_. If this is so, many similarities between humans and chimpanzees would represent homoplasies rather than homologies. While such a high rate of homoplasy may seem unlikely, Duda and Zrzavý (2013) report rates of homoplasy for their most parsimonious tree that are within the range of other biological datasets.

In the absence of additional evidence, such as fossils or a more detailed understanding of how genes relate to traits, reconstructions of the LCA_PH_ are necessarily speculative. Moreover, even when a good fossil record is available, much remains unknown about soft tissue and behaviour. An improved fossil record for African apes in the late Miocene would help resolve some of these questions. Additionally, genetic studies may shed light on the evolution of traits such as exaggerated sexual swellings.

## Why apes, not monkeys, became human

If many of the similarities between chimpanzees and humans result from homoplasy rather than homology, what is the use of comparing chimpanzees to humans? Fuentes (2018) argues that ‘what chimpanzees and humans do today is not directly comparable – because we have evolved independently for millions of years’. However, comparison of traits among diverse lineages is a central tool in evolutionary studies; the comparative method is one of Darwin's many valuable gifts to science.

We did not evolve from chimpanzees, but we did evolve from something that was more like a chimpanzee than it was like human (Begun, 2016). Our last common ancestor with chimpanzees surely didn't have language, or cooking, or habitual bipedal locomotion. If we want to understand why those traits evolved in the hominin lineage, the best way to test hypotheses is to study our modern relatives: chimpanzees, bonobos, gorillas and other primates. Not every trait, of course, is best studied in our closest relatives; a broad view of biology is needed to benefit fully from the comparative method. The dance communication of bees, for example, is more similar to human language in some respects than any non-human primate communication system (Section 4.7). However, for many traits, it matters that we evolved from a specific kind of animal: a large-bodied, orthograde, ripe fruit specialist ape.

### Adaptive landscapes

**Figure 2.**
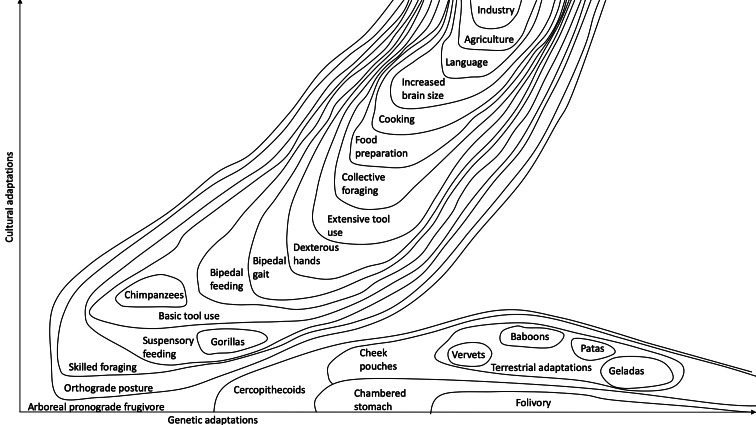
Adaptive landscape for African apes and monkeys.

To illustrate the peculiar path that hominin evolution has followed, and how it depends on our ape ancestry, Figure 2 depicts an adaptive landscape for selected African monkeys and apes, following the metaphor of Wright (1932). The *x*-axis represents variation in genetic space (which is vastly multidimensional, but simplified here for the sake of illustration). Moving to the right along this axis represents increasing genetic adaptation to environmental challenges, while the *y*-axis represents variation in cultural space, which is also vastly multidimensional, but simplified here as a single axis, representing socially learned adaptations to environmental challenges. The contour lines represent fitness. Both genetic and cultural traits increase in frequency within populations if they produce a net beneficial effect on the carrier's inclusive fitness. Over time, this causes populations to move uphill, finding nearby fitness peaks. Arrows drawn perpendicular to the contour lines would represent vectors: directions in which natural selection tends to move populations. The figure necessarily represents a static landscape, but in reality this landscape is dynamic, shifting as climate change, newly evolved species and cultural innovations create new challenges and opportunities. Research at Gombe has provided insights into some possible pathways across this adaptive landscape.

### Diet

Diet drives adaptations: ‘you are what you eat’. We therefore begin our tour of the primate adaptive landscape by focusing on dietary adaptations.

Goodall (1963) found that chimpanzees eat a broad range of items: leaves, fruit, flowers, seeds, pith, gums, insects, honey and a variety of smaller mammals, particularly monkeys. Further study revealed that, within this broad diet, chimpanzees, like other apes, prefer ripe fruit (Wrangham, 1977).

**Figure 3.**
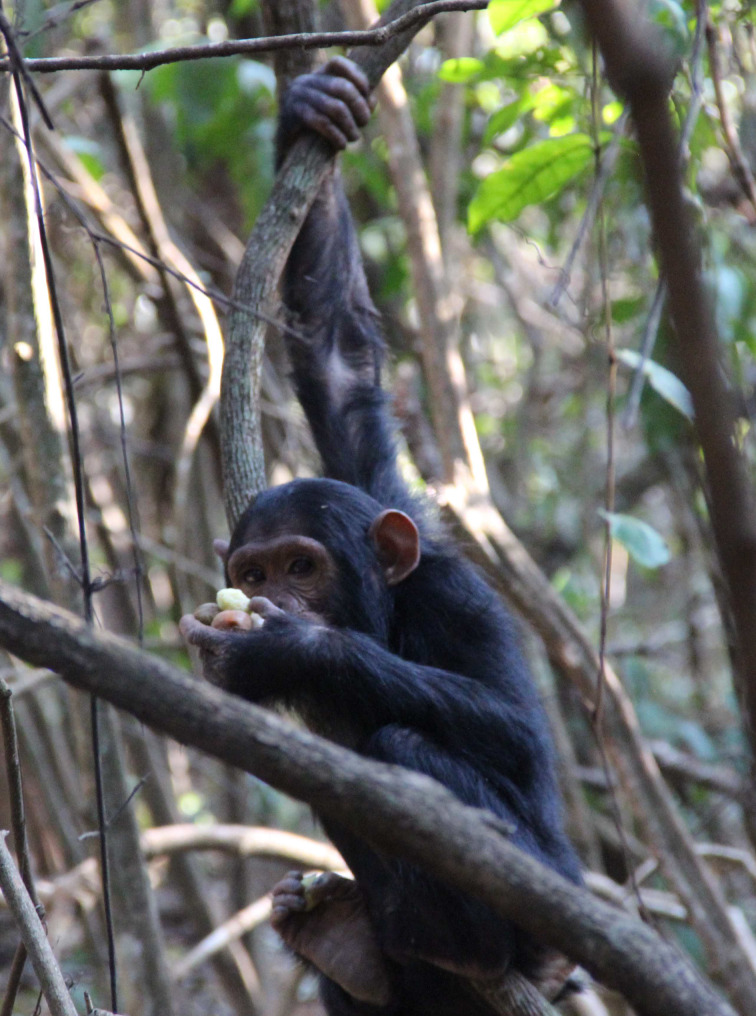
Kasekela juvenile male Fede using suspensory feeding posture, holding fruits of *Parinari curatellifolia* in hand and foot. Photo by Michael L. Wilson.

Fossil evidence indicates that the ancestor of catarrhines – apes and Old World monkeys – was probably an arboreal frugivore with the pronograde (horizontal) body posture typical of mammals (Begun, 2016). Studies of dentition, microwear and reconstructions of habitat indicate that apes have long specialized in eating ripe fruit (Potts, 2004). Tooth shape indicates that early Miocene apes probably relied on fruit to an even greater extent than present-day apes (Potts, 2004). Apes evolved large body size and an orthograde (upright) feeding posture, and lost their tails, which are less necessary when apes climb deliberately and hang from branches, rather than scampering along the tops of branches, and also less effective in balancing large body masses. Modern apes have suspensory adaptations (freely rotating arms, narrow shoulders and short, stiff backs), which enable them to gain access to fruits on terminal branches too small to support their weight (Hunt, 2016; Figure 3).

Apes once ranged widely across Eurasia as well as Africa, reaching a peak in species diversity in the Miocene, between 11 and 9.5 Ma (Potts, 2004). After this time, the global climate became cooler and drier. Ape diversity declined, and the range of apes contracted towards the equator, tracking the contracting range of moist forests. Begun (2016) views at least some of these European apes as having had suspensory feeding adaptations, and to have been close to the ancestry of crown hominoids such as gorillas and chimpanzees.

Modern African apes occupy distinct adaptive peaks. Gorillas evolved large body size, providing the hindgut capacity needed to ferment herbs effectively. This enables them to survive on mountains with few fruit trees, but it also traps them on a narrow fitness peak: moist forests close to the equator, with a year-round supply of readily digestible fruits and herbs. Bonobos likewise appear best adapted for moist forest; even in forest–savanna mosaics, they prefer mature forest for feeding and nesting (Serckx et al., 2015). Chimpanzees live across a broader swath of Africa, from Senegal to Tanzania, from the rainforests of the Congo basin to the arid savannas of Mali. Cultural adaptations help chimpanzees survive in these varied habitats, using stones to crack nuts, sticks to dig for tubers and spear bushbabies and hands to dig wells, and sheltering from the heat in pools and caves (McGrew, 2010). Chimpanzees have thus shifted further up the cultural dimension (Figure 2).

Cercopithecoid monkeys have followed a different trajectory from apes – moving to the right along the genetic axis through a series of dietary adaptations (Figure 2). Colobine monkeys evolved a cow-like chambered stomach that enables them to digest a broad range of foods that are unpalatable to apes, such as mature leaves. Cercopithecine monkeys evolved cheek pouches, where amylase-secreting glands break down the starch of unripe fruit into more readily digestible sugars (Lambert, 2005). Controlling for body size, cercopithecine monkeys also retain food in their digestive tracts for a longer period (Lambert, 2007).

These physiological adaptations have enabled monkeys to thrive in habitats unsuitable for apes. As Europe became cooler, drier and more seasonal in the late Miocene, apes declined to extinction, while monkeys diversified (Potts, 2004). Monkeys maintained the ancestral pronograde posture, making them efficient quadrupeds on the ground as well as in trees. As forests contracted in Africa, monkeys found multiple pathways to exploit open woodlands and savannas, represented by a range of peaks occupied by savanna monkeys (vervets and their allies: *Chlorocebus* spp.), patas monkeys (*Erythrocebus patas*), geladas (*Theropithecus gelada*) and baboons (*Papio* spp.). Savanna monkeys show the least obvious degree of genetic adaptation, with bodies closely resembling those of their cousins, the forest guenons (*Cercopihtecus* spp.; Selby, 2018). Patas monkeys have evolved long limbs and fleet bodies like greyhounds, enabling them to flee predators and travel efficiently among widely dispersed food resources (Selby, 2018). Baboons have evolved large body size and broad diets, but are also highly selective feeders (Barton & Whiten, 1994), and are the most widely distributed of African primates, besides humans. The one remaining species of gelada (*Theropithecus gelada*) is a specialized graminivore, eating mainly grass and herbs (Fashing et al., 2014). Genetic adaptations can lead populations to fitness peaks that make cultural innovation less likely; geladas do not need tools, for example, when they can graze in the meadows.

**Figure 4.**
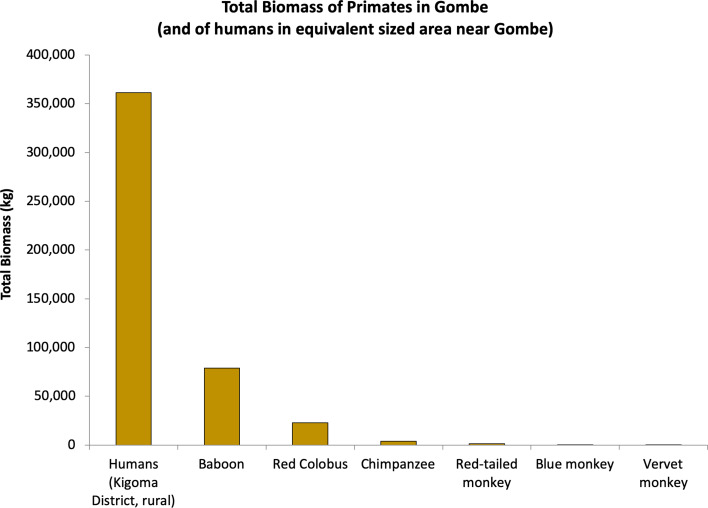
Estimated biomass of primates in Gombe (and of humans in an equivalent sized area near Gombe). Human population is calculated for areas *outside* the park, based on population density in rural Kigoma District (218.6 people/km^2^); mean adult body mass of 60.7 kg, (Walpole et al., 2012); 44% of Tanzanian population under 15 (CIA World Factbook); and assuming that everyone above 15 years old is average adult body mass, and everyone below 15 is half that. Non-human primate data from Falk Grossman (unpublished data).

Apes lack the dental and physiological specializations that enable monkeys to subsist on the coarser foods that are readily available in more seasonal habitats. As Byrne (2016) argues, apes evolved skilled foraging methods, including tool use, to compete with monkeys. Even so, monkeys have reached higher adaptive peaks than apes, even in forests. Monkeys outmass chimpanzees at Gombe by a factor of nearly 26 (Figure 4), and similarly outmass apes at other African field sites (Lambert, 2007).

Humans followed a narrow and winding path up a mountain that towers over all the other primate peaks. In the rural landscape of Kigoma District, surrounding Gombe National Park, the human population on an average piece of Gombe-sized land outmasses the entire primate population at Gombe more than three times (Figure 4). Globally, humans and our livestock constituted an estimated 97.2% of the biomass of land mammals in 2000 (Smil, 2011).

The pathway to this high peak depends on a series of both genetic and cultural adaptations. In many cases, cultural innovations created selection pressures for new genetic changes, and vice-versa, through gene–culture co-evolution (Richerson & Boyd, 2004; Henrich 2017). Insofar as apes learn to forage from their mothers, the suite of foods they learn to use represents cultural knowledge. Apes faced with drying habitats and shrinking forests can either retreat along with the forest or learn to acquire foods in more open landscapes. Cultural changes, such as learning to use foods that require a bipedal feeding posture, probably promoted genetic changes for improved bipedal posture, which increasingly freed the hands to make and use tools (Darwin, 1871). Tools and cumulative culture enabled hominins to thrive in new habitats (Richerson & Boyd, 2004; Henrich 2017). Using tools and fire to smash, grind and soften foods reduced the size of teeth, jaws and guts while providing energy to grow larger bodies and brains (Wrangham, 2011). Increasing brain size promoted the co-evolution of brains and symbolic communication (Deacon 1997). Such gene-culture co-evolution moved hominins both upwards and rightwards along the ridge, leading towards the current human fitness peak.

### Tools

Human subsistence relies on cultural adaptations: weapons for hunting, knives for butchering, baskets for gathering, pots for cooking, and clothing and shelter for protection from the elements. The rapid speed of cultural evolution enabled hunter–gatherers to expand into nearly all terrestrial habitats, despite only modest changes in biological adaptations (Henrich, 2017). Complex tools such as hafted spears, baskets and canoes emerge through many generations of cumulative culture, in which the accumulating insights and innovations of thousands of ancestors contribute the know-how necessary to make items that would defy the powers of a lone individual (Boyd et al., 2011). Chimpanzees do not have cumulative culture, but research at Gombe and other sites has revealed that they make and use tools, and exhibit cultural traditions, which provides a provocative model for how material culture may have emerged in early hominins.

Tools have been a major focus of research at Gombe since the beginning. American tool manufacturer Leighton Wilkie supported Leakey's work on stone tools at Olduvai, and also provided the initial funding for Goodall's study (Peterson, 2006). On 4 November 1960, Jonathan and Mary Leakey discovered OH7, the type specimen for *Homo habilis*, whose name (‘handy man’) represented Leakey's conviction that the maker of the Oldowan tools had finally been found. That same day – in the final month of Goodall's initial study – Goodall observed the chimpanzee David Greybeard fishing for termites (Peterson, 2006). Goodall soon observed chimpanzees fashion wands for termite fishing, by removing leaves from a stem. This confirmation that chimpanzees make and use tools proved key to attracting funding from the National Geographic Society that extended research at Gombe into a truly long-term study.

Tool use has remained a major focus of research at Gombe (e.g. Lonsdorf et al., 2004; McGrew, 1979; Musgrave et al., 2020; O'Malley et al., 2012; Teleki, 1974; Whiten et al., 1999). Tool use gives chimpanzees access to easily digestible, energy-rich foods such as social insects, nuts, honey and brains. As Byrne (2016) notes, baboons eagerly eat termites when swarms of reproductive alates emerge from their nests, but lack tools to extract them from their nests. In contrast, chimpanzees at Gombe consume termites extensively, particularly during the wet season, when rain softens the outside of the mounds, making it easier to insert tools. Chimpanzees also use tools to eat driver ants (*Dorylus* spp.), which are rich in protein (O'Malley & Power, 2014), but whose fierce bites dissuade most potential predators. Chimpanzees prey on these ants by ‘dipping’ for them, inserting a wand made of vegetation into a column of ants or the entrance of their nest (McGrew, 1979). The ants bite the wand with their mandibles – making them both easy to transport to the mouth and safe to consume.

**Figure 5.**
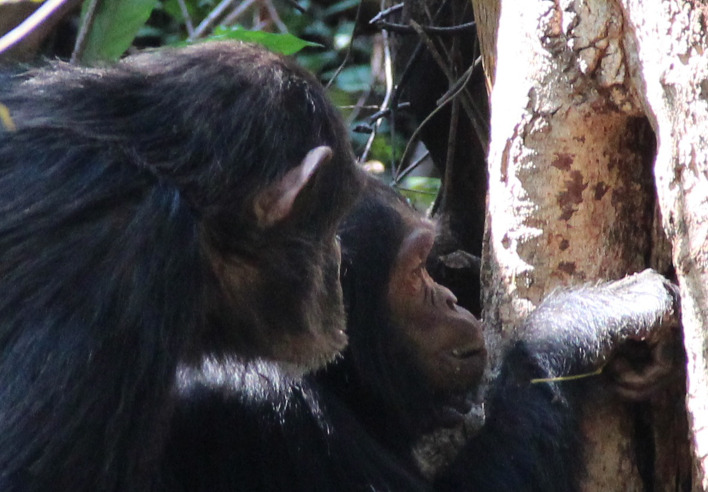
Flirt, a female immigrant to Mitumba from the Kasekela community, watching Mitumba-born female Flower fishing for ants (probably *Camponotus* spp.). This technique is habitual in Mitumba, and appears to have spread to Kasekela with immigrant female Trezia in the 1990s (O'Malley et al., 2012). Photo by Michael L. Wilson.

Observations at Gombe suggested that females using perishable tools to acquire insect and plant foods played an important role in hominin evolution, in contrast to the once prevailing focus on stone tools and hunting (Zihlman, 1978; McGrew, 1979). Gombe females spend more time than males termite fishing (Pandolfi et al., 2003) and ant-dipping (McGrew, 1979). Lonsdorf and colleagues (2004) found that young females gain proficiency at tool use faster than young males. Similarly, at Taï, Côte d'Ivoire, females spend more time than males cracking nuts (Boesch-Achermann & Boesch, 1993). O'Malley et al. (2012) report evidence that, when females transfer among communities, they may bring with them new cultural knowledge (Figure 5). However, at Taï, group differences in nut-cracking patterns persisted despite females transferring among neighbouring communities (Luncz & Boesch, 2014).

The extent to which the LCA_PH_ made and used tools may never be known, as the tools used by chimpanzees leave only scant evidence for archaeologists. Neither gorillas nor bonobos use tools to any great extent in the wild, so perhaps extensive tool use evolved convergently in humans and chimpanzees. Nonetheless, the ape requirement for high-quality food appears to be a major factor promoting tool use in both lineages.

Tools may have been important for making possible the dietary shifts revealed by stable isotope studies. Analysis of the ratios of stable isotopes of carbon in hominin fossils has revealed a striking shift in the diets of hominins, away from C_3_ plants – forest trees, shrubs, lianas and herbs – towards increasing use of C_4_ plants: grasses and sedges (Cerling et al., 2011; Nelson & Hamilton, 2017; Sponheimer et al., 2013). Nockerts (2019) analysed stable isotope signatures of chimpanzees, baboons and their foods at Gombe. Examining these new data in context with published data on chimpanzee, baboon and hominin isotope signatures, Nockerts has found that even early hominins had distinct stable isotope signatures from chimpanzees, including ‘savanna’ chimpanzees (Nockerts, 2019).

As apes, how did hominins gain access to this diet, without the physiological adaptations of monkeys? Presumably hominin diets differed substantially from those of baboons, even if their stable isotope signatures indicate similar consumption of C_4_ resources. The teeth and jaws of australopiths differ greatly from those of baboons – although they are similar in some respects to those of geladas (Jolly, 1970). Underground storage organs of plants – roots and tubers – have been proposed as a major fallback food for australopiths (Laden & Wrangham, 2005). Hunter–gatherers use digging sticks to gain access to these resources. Chimpanzees in western Tanzania, in the dry, open habitat of Masito–Ugalla, have been found to do this as well (Hernandez-Aguilar et al., 2007). Simple tools that chimpanzees are capable of using thus may have been a key factor in enabling early hominins to adapt to more open habitats.

### Bipedal gait

Darwin (1871) inferred that bipedal locomotion was a key early step in human evolution. The reasons why bipedal locomotion evolved, however, remain unclear. Many hypotheses presume that bipedal gait evolved in response to increasing use of more open habitats. As noted above, monkeys have adapted to open habitats multiple times, but none have evolved bipedal gait. Why did the ancestors of humans follow a different path? One likely factor is that monkeys were already well adapted to quadrupedal locomotion. When in trees, they walk quadrupedally along the tops of limbs (Selby, 2018). Apes, in contrast, have evolved an orthograde posture for feeding and climbing (Selby, 2018). Adaptations for manoeuvring their large bodies in trees have made apes inefficient quadrupeds on the ground.

Data from Gombe and other sites indicate that chimpanzees travel shorter distances each day than human foragers. A study of the Hadza hunter–gatherers in Tanzania found that mean daily walking distances were 5.8 km/day for women and 11.4 km/day for men (Pontzer et al., 2012), much greater than the daily travel distances for chimpanzees at Kanyawara (females 2.0 km/day; males 2.4 km/day) or Gombe (females 3.2 km/day; males 4.6 km/day; Pontzer & Wrangham, 2004). Baboons, on the other hand, travel further each day than chimpanzees, and in some cases rival the distances travelled by human foragers. Yellow baboons in Amboseli, Kenya travel about 5.5 km/day, while hamadryas baboons travel an average of 13.2 km/day (Altmann, 1974).

Rodman and McHenry (1980) proposed that bipedal locomotion enabled early hominins to travel more efficiently between dispersed food patches, while also retaining the forelimb anatomy needed to climb into feeding trees. Consistent with this hypothesis, laboratory studies have found that humans walk more much more efficiently than chimpanzees (Sockol et al., 2007). The first steps onto the narrow and winding path towards hominization thus probably resulted from our ape ancestors being better adapted for climbing than walking.

Laboratory studies have found that chimpanzees are equally inefficient at bipedal and quadrupedal locomotion (Pontzer et al., 2014). Bipedal locomotion therefore seems unlikely to have increased efficiency of travel in the earliest hominins. Adaptation for efficient bipedal locomotion seems most likely to have occurred if it followed an earlier phase of selection for increased use of bipedal posture. The wading hypothesis, inspired by observations of baboons foraging among islands in the Okavango delta (Wrangham et al., 2009), proposes that water provided the support necessary to stabilize bipedal posture in early hominins. Alternatively, the postural feeding hypothesis, based on observations of geladas, suggests a source of selection pressure for feeding in drier habitats (Wrangham, 1980).

**Figure 6.**
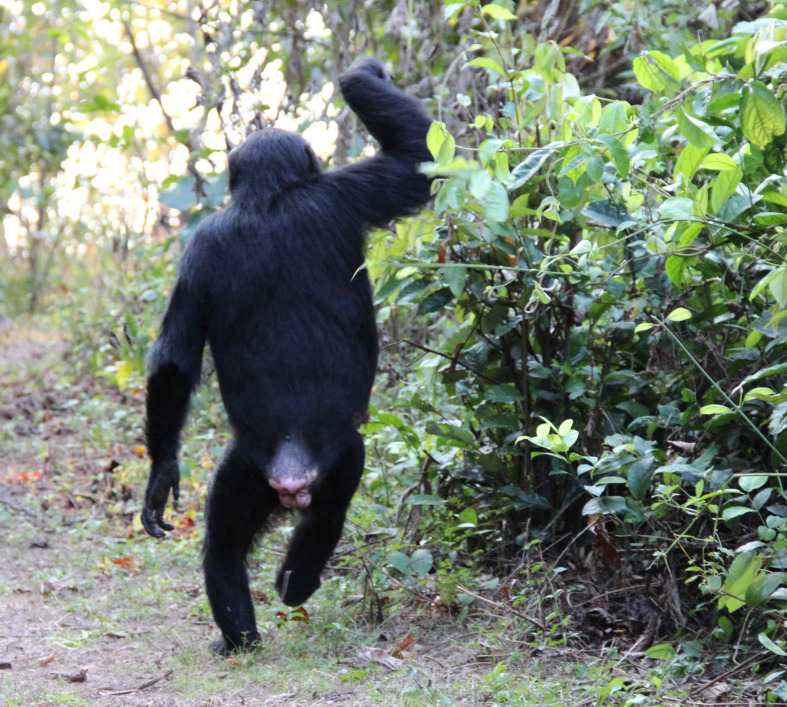
Kasekela female Imani standing bipedally to feed from low vegetation. Photo by Michael L. Wilson.

Because primates spend much of each day feeding, feeding posture is likely to provide an important source of selection pressure, and a mechanism by which increased bipedality would benefit early hominins before they became efficient walkers. In a study based at both Gombe and Mahale, Hunt (1994) found that chimpanzees and baboons engaged in bipedal posture most commonly when feeding (Figure 6). Hunt argued that early hominins stood bipedally to forage on small objects from small trees and shrubs, which are common in open habitats. Consistent with this hypothesis, Tourkakis (2009) found that chimpanzees in the dry, open habitat of Fongoli, Senegal engaged in more bipedal standing when feeding, as well as more bipedal locomotion, compared with chimpanzees at Gombe and other less open habitats.

### Multilevel societies

Field studies of apes have provided valuable insights into the evolution of human societies. Chimpanzee societies resemble human societies in having what Kummer (1971) called ‘fusion–fission’ grouping patterns: members of a group travel in subgroups of varying size, in contrast to the stable groups of gorillas and many other primates. Chimpanzees differ from humans, however, in not having stable pair-bonds between males and females, and in not having higher-level patterns of affiliation – features that lead researchers to classify humans, but not chimpanzees, as having multilevel societies (Grueter et al., 2012).

It took over a decade of research for researchers to gain a clear picture of chimpanzee social structure at Gombe (Goodall et al., 1979). As Goodall learned to recognize individuals in her first years at Gombe, she observed that chimpanzees travelled in subgroups (‘parties’) that varied in size and composition; the only stable grouping appeared to be mothers and their offspring (van Lawick-Goodall, 1968a). Because different parties joined together without aggression, Goodall initially speculated that chimpanzees lived in unbounded societies, which she called ‘communities’ (Goodall et al., 1979). Nishida, who began working at Mahale in 1965, more quickly determined that chimpanzees live in closed social groups (Nishida, 1968). Two key factors helped Nishida gain this understanding. First, the location where Nishida planted sugarcane to attract chimpanzees happened to be located in the boundary area between two groups, enabling him to observe intergroup interactions early on (Nishida, 2012). In contrast, Goodall's feeding station at Gombe was located near the centre of the study group's range; research for much of the 1960s focused on behaviour in and around the feeding station, making observation of intergroup encounters unlikely. Second, Nishida came to Mahale specifically seeking to understand chimpanzee social structure (Nishida, 2012). His mentors, Imanishi and Itani, shared a strong interest in the evolutionary origins of human societies (Matsuzawa & McGrew, 2008). Imanishi initially reasoned that chimpanzees, like gorillas, might live in one-male groups, which he called ‘familoids’, thought to provide an evolutionary precursor to the human family (Imanishi, 1965). His student Itani later speculated that chimpanzee groups might consist of multiple familoid groups (Itani, 1966). Nishida found no evidence for familoid groups; instead, like at Gombe, Mahale chimpanzees travelled in extremely fluid parties. Consistent with observations suggesting closed social groups at Filabanga, Tanzania (Itani & Suzuki, 1967), Nishida found that Mahale chimpanzees associated with one of two distinct groups: Kajabara and Mimikire (Nishida, 1968). Mimikire had more members and appeared socially dominant (Nishida, 1968). Nishida called these bounded societies ‘unit-groups’ (Nishida, 1968).

At Gombe, the existence of social boundaries became apparent by the late 1960s (van Lawick-Goodall, 1971), and became strongly evident in the early 1970s, when the study group split into the mutually hostile Kasekela and Kahama communities (Goodall et al., 1979). Intergroup killings subsequently resulted in the Kahama community's extinction (Goodall, 1986). Goodall and other Gombe researchers continued to use the term ‘community’ to refer to chimpanzee groups, but shifted the meaning, making it synonymous with ‘unit-group’. These two terms persist in the literature, with European and American researchers tending to use ‘community’, while Japanese researchers tend to use ‘unit-group’ (Itoh & Nakamura, 2015). Nishida avoided using the word ‘community’ in this sense, not only because ‘unit-group’ referred specifically to closed social groups, but also because Imanishi used ‘community’ to describe a characteristic feature of human societies: ‘social units do not exist independently but are integrated into a higher unit’ (Nishida, 2012: 3). Imanishi's understanding of ‘community’ thus appears equivalent to the term ‘multilevel society’.

The multilevel societies of humans consist of families with long-term breeding bonds that identify as members of larger groupings, such as bands, ethno-linguistic groups, tribes and nations (Grueter et al., 2012; Wilson & Glowacki, 2017). None of the other apes have a clearly multilevel society, although aggregations of groups of western gorillas, and gatherings of parties from different bonobo communities, are similar in some ways (Furuichi, 2020). The multilevel societies of gelada monkeys and hamadryas baboons provide better analogues of this aspect of human societies.

Why do these monkeys live in multilevel societies? Two factors appear to be key: food and sleeping sites (Grueter et al., 2012). Monkeys are able to find sufficient food to forage in stable groups under a broad range of conditions, but in the arid lands of the Horn of Africa and Arabia, even baboons are forced to disperse into smaller foraging parties, at least in some seasons. Geladas live at high elevation, where there is more rainfall, but seasonal variation in food availability forces large herds to scatter into smaller groups. The second factor promoting multilevel societies appears to be the scarcity of safe sleeping sites. In forested areas, primates can easily find trees to sleep in safely away from predators. In more open country, suitable refuges are sparse, consisting mainly of groves of trees along watercourses and cliffs. Geladas and hamadryas baboons both rely mainly on cliffs. Cliffs suitable for sleeping sites are commonly large enough to accommodate many individuals, and so serve as common aggregation points at the end of the day for parties that have been foraging separately. A small group cannot readily monopolize a sleeping site with room for many groups, and so scarce but capacious sleeping sites will readily be shared by multiple groups.

Given that fossils of hominins, including *Australopithecus* and *Ardipithecus*, have been found in the region now inhabited by hamadryas baboons, it seems plausible that these hominins would have sought refuge on cliffs at night, and dispersed throughout the day in smaller foraging parties. As Marlowe (2005) has noted, women in present-day hunter–gatherer societies such as the Hadza forage for many of the same resources hypothesized to be important for early hominins (berries and nuts from trees and bushes, underground storage organs), and technology such as digging sticks would also probably have been available to early hominins. Given that Hadza women generally forage in groups of about five (Marlowe, 2005), it seems plausible that earlier hominins would have been able to do so as well.

The long-term breeding bonds that characterize multilevel societies may result from females foraging in smaller groups, which makes fertile females easier for males to monopolize (Grueter et al., 2012). Simulations using agent-based computer models support this hypothesis (Crouse et al., 2019). Such stable breeding bonds differ strikingly from the more promiscuous mating patterns of *Pan*. If the LCA_PH_ had *Pan*-like societies, then stable breeding bonds may have emerged from a more promiscuous mating system, as probably happened in hamadryas baboons and perhaps geladas as well. If the LCA_PH_ instead had gorilla-like one-male societies, then stable breeding bonds would be an ancestral trait for hominins. In this case, multilevel societies would have evolved from the aggregation of smaller units, rather than the disintegration of troops.

If hominins regularly used a small number of regular sleeping sites, this could promote the evolution of another distinctive hominin trait: central place foraging. Any foods that required more extensive processing before eating might be carried back to the sleeping cliffs and worked on there. Any food preparation at a central site would be visible to other group members, and so might serve as an impetus to sharing (and/or scrounging).

### Collective foraging

The human subsistence strategy – hunting and gathering – differs profoundly from that of any living primates, with its sexual division of labour (men do most of the hunting, women do most of the gathering) and transfers of food (Isaac, 1978). Hunting and fishing account for over half of the daily energy obtained across foraging societies (Kaplan et al., 2000), providing humans with food in habitats where apes would starve, such as climates where arid conditions or long winters make ripe fruit unavailable. Gathering differs from the foraging of chimpanzees and other primates in three major ways: (a) gatherers bring much of what they acquire back to a central camp, where they (b) normally prepare the food to make it more palatable and (c) share it with others. Food transfers from parents and others to offspring promote offspring survival, shorten interbirth intervals and enable human populations to reproduce more quickly than other apes (Kaplan et al., 2000). In contrast, other primates mainly eat as they go, and fend for themselves, rather than collecting food to share. While chimpanzees do not engage in full-scale collective foraging, studies at Gombe and other sites have revealed that chimpanzees do engage in some sharing of food, providing insights into how collective foraging may have evolved in the human lineage.

On 30 October 1960, Goodall observed David Greybeard eating an infant bushpig, and sharing the meat with a female. Hunting and meat sharing have been major research topics at Gombe ever since (Boesch, 1994; Stanford et al., 1994; Gilby et al, 2015; Goodall, 1963; Teleki, 1973).

Hunting presents an interesting problem for cooperation. Hunters incur energetic costs and risk of injury in pursuing prey. Individuals can cheat by opting out of the hunt, but obtaining meat afterwards, by either theft or begging. Two solutions have been proposed: reciprocity (hunters share meat more with other hunters, and/or with key allies and/or fertile females) and self-interest (males hunt if they are highly motivated to acquire meat for themselves, and they share meat to reduce harassment from beggars).

Boesch (1994) argued that hunting at Taï is maintained by reciprocity: hunters share more meat with other hunters than with bystanders. In contrast, Wrangham (1975) proposed that chimpanzee sharing is more selfish: chimpanzees share to induce beggars to leave them alone so that they can feed more efficiently. Detailed analysis of video from meat-sharing events confirmed key predictions of Wrangham's hypothesis: more persistent beggars obtained more food; beggars left after getting food; and the carcass-holder's feeding efficiency increased when fewer beggars were present (Gilby, 2006; Figure 8).

**Figure 7.**
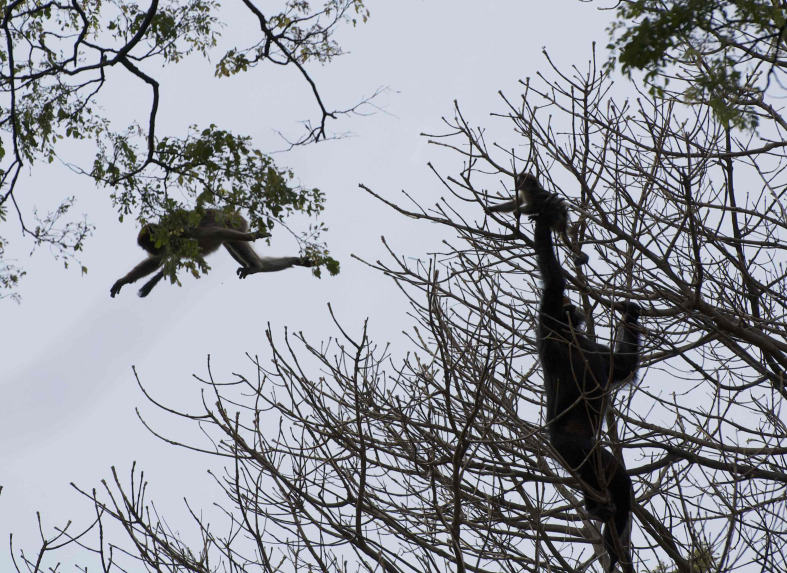
Adult red colobus monkey leaping across a gap in the canopy to escape from a female chimpanzee, Samwise, who is grabbing a juvenile colobus. Photo by Ian C. Gilby.

Boesch visited Gombe in the early 1990s and speculated that ecological differences influenced patterns of cooperation, with cooperation being more critical for success in the high canopied forest of Taï (Boesch, 1994). This hypothesis has not been tested systematically, but Gilby et al. (2006) confirmed Wrangham's (1975) observation that Gombe chimpanzees were more likely to hunt when in woodlands – where the broken canopy made it more difficult for monkeys to escape – than in evergreen forest (Figure 7).

**Figure 8.**
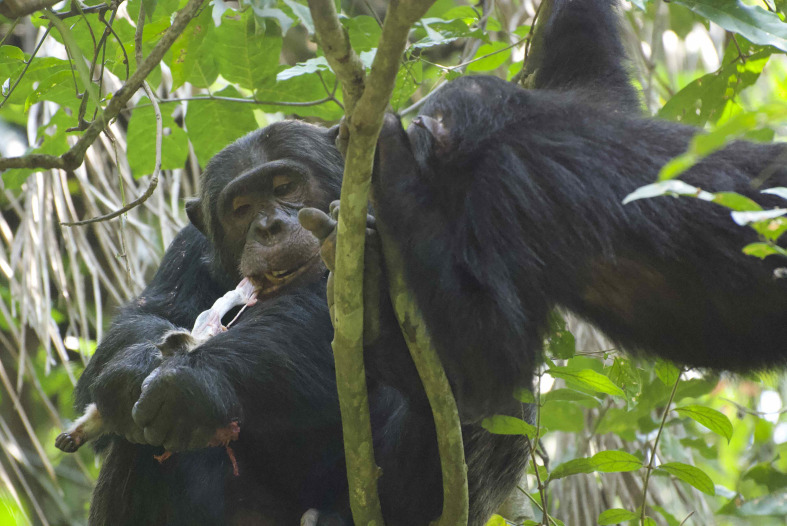
Edgar, an adult male in the Mitumba community, eating a red colobus monkey, with adolescent female Yamaha watching. Photo by Ian C. Gilby.

Stanford argued that male chimpanzees hunt to obtain meat to share with fertile females, based on observations that encounters with red colobus monkeys led to hunting attempts more often when sexually receptive females were present (Stanford et al., 1994). This suggested an intriguing analogy to food transfers in hunter–gatherers (Stanford, 1996). However, subsequent analysis of a larger dataset from Gombe, which included the years Stanford analysed, failed to support this hypothesis (Gilby et al., 2006). Instead, the presence of receptive females made males less likely to hunt – presumably because males can invest either in hunting or in mate guarding, but not both at once. Analysis of data from both Kanyawara and Gombe found that males neither shared preferentially with receptive females nor increased their mating success by sharing with receptive females (Gilby et al., 2010). While female chimpanzees are eager to obtain meat, they do not require meat as an inducement to mate (Gilby et al., 2010). Stanford's results may have differed from later analyses because during the years Stanford analysed, the sterile female Gigi was often present, and often swollen. Male chimpanzees show substantially greater interest in mating with females that have produced offspring (Muller et al., 2006), so the presence of Gigi may have provided little distraction from an interest in hunting.

Observations that hunting success increases with male party size appear to support the view that chimpanzee hunting is a cooperative endeavour (Boesch, 1994; Stanford et al., 1994). Gilby and colleagues have found, however, that this effect depends on the presence of a small number of key individuals: ‘impact hunters’. If an impact hunter is present, larger parties are more likely to hunt, but in the absence of these individuals, hunts are unlikely to occur (Gilby et al., 2015). Presumably, the risks and costs of hunting are highest for the first individual to start the hunt, but once a hunt begins, others stand to gain from participation. Hunting in chimpanzees thus appears to be a byproduct of mutualism.

Hunting was a key factor enabling hominins to navigate their adaptive landscape, climbing higher up the narrow ridge towards distinctively human adaptive peaks. Whether hominins followed that path because their ancestors hunted remains uncertain. If the LCA_PH_ lived in single male groups, opportunities for hunting by multiple males would have been rare. Even if the LCA_PH_ closely resembled chimpanzees, any hunting conducted by hominins probably differed from the chimpanzee pattern. As McGrew (2010) observes ‘Most chimpanzee hunting is done arboreally, by “four-handed” hunters who can leap about in the canopy, pursuing monkeys. This is not likely to be instructive about hunting by terrestrial bipeds’. Human hunting depends on bipedal locomotion – which enables hunters to travel long distances efficiently in the pursuit of prey, and frees the hands to make and use weapons and carry meat back to camp – and cumulative culture, which preserves and transmits innovations for capturing, killing and processing prey. The evolution of extensive hunting in hominins might therefore depend more on the various factors that led to bipedal locomotion and cumulative culture than on a heritage of hunting. Nonetheless, hunting and meat sharing in hominins would have posed similar problems for cooperation. Field studies of chimpanzees provide insights into how these problems can be solved.

Observations of chimpanzees suggest that, long before hominins shared meat on a large scale, mothers shared plant foods with their offspring (McGrew, 1979). Chimpanzee mothers commonly share foods that are difficult to open, such as *Strychnos* and *Diplorhynchus*, with their offspring (Silk, 1978; McGrew, 1979). At Taï, where chimpanzees use stones and anvils to crack nuts, mothers allow their offspring to take nuts they have opened (Boesch-Achermann and Boesch, 1993). When technology enables individuals to produce food surpluses, mothers have strong incentives to share food with their offspring, both to promote the growth and survival of their offspring and to offset their own lactation costs (Silk, 1978). Such food sharing by mothers may have played an important role in the evolution of more cooperative temperaments, by promoting an increased capacity to inhibit impulses and keep in mind the needs of others.

### Language

Language so completely permeates human life that fully understanding what it enables us to do, and what can be done without it, can be difficult. Language shapes our thinking and imagination. It helps cooperation, enabling individuals to share detailed, explicit plans and to negotiate coalitions and trade. It provides a conduit for cumulative culture, storing and transmitting the stories, knowledge and know-how of our ancestors and distant others who we will never meet. The reasons for how and why human language evolved remain unclear, but research in diverse fields suggests that an empirically grounded, comparative approach can shed light on these questions (Fitch, 2010).

Because chimpanzees are so similar to humans in many respects, researchers have sought evidence of language-like capacities in chimpanzee vocal communication (Fedurek & Slocombe, 2011). Marler recorded chimpanzee vocalizations at Gombe in 1967, and based on acoustic analysis, identified a repertoire of 13 basic types, which generally resembled those of gorillas (Marler, 1976). Goodall (1986) identified a richer set of 32 categories that sounded distinct to her. Classifying chimpanzee calls is challenging, because many categories grade into one another. Crockford (2019) proposed a hierarchical system that classifies most chimpanzee calls as variants of five basic categories: screams, barks, grunts, hoos and pants.

Marler's work inspired two main avenues of searches for language parallels: referential signalling and dialects. Marler (1977) considered food-associated ‘rough grunt’ calls to be the most promising candidates for referential signalling. Slocombe and Zuberbühler (2005) proposed that different categories of rough grunt signalled food preference: high-pitched, more tonal calls were given in response to more preferred foods, while low-pitched, noisier calls were given in response to less preferred foods. However, field studies in Budongo (Slocombe & Zuberbuhler, 2006) and Taï (Kalan et al., 2015) have found only limited evidence for associations between acoustic structure and food characteristics. At Gombe, O'Bryan (2015) found that acoustic features varied considerably within bouts, indicating that these calls have low potential for referential signalling. These calls may instead signal more about the caller's intentions to continue feeding (Fedurek & Slocombe. 2013; O'Bryan, 2015).

Chimpanzees give acoustically distinct alarm calls to snakes, which appear to be better candidates for functionally referential calls (Crockford et al., 2015). An intriguing possibility suggested by long-term researchers at Gombe is that chimpanzees have distinct calls for pythons and cobras.

Dialects – regional variation in acoustic structure – are thought to reflect vocal learning, which is a key component of human speech, but a rare trait in mammals. Mitani compared recordings of pant-hoots from Mahale with Marler's recordings from Gombe, and found evidence for dialects (Mitani et al., 1992). However, Mitani later argued that the evidence for vocal learning in chimpanzees was slim: most variation in acoustic structure depended on individual identity, rather than community membership (Mitani & Brandt, 1994). Subsequent studies have found evidence for dialects at Taï (Crockford et al., 2004). Work is currently underway to assess whether the Mitumba and Kasekela communities at Gombe have distinct pant-hoot calls; preliminary analyses indicate that they do not (Desai & Wilson, 2020).

Chimpanzees also produce many gestures (van Lawick-Goodall, 1968b). Gestures have long been of interest as a possible step along the path towards language, particularly given that apes appear to have more intentional control over their gestures than their vocalizations (Pollick & de Waal, 2007). Perhaps challenging the view that gestures represent learned signals, however, the physical forms of gestures used by chimpanzees at Budongo, Uganda and bonobos at Wamba, overlap by approximately 90% in their physical shape, and to a considerable extent, gestures of the same form share the same apparent meaning in the two species (Graham et al., 2018).

The modest evidence for referential communication and vocal learning in chimpanzees suggests that the key events in language evolution occurred only in the hominin lineage, after the divergence from panins. More recent efforts to find language parallels have focused on aspects of social cognition, which surely are relevant for understanding language evolution (Crockford et al., 2017). Nonetheless, considering the capacity to accurately inform others about remote objects, the closest parallel to human language comes not from chimpanzees or bonobos or any other primate, but instead from social insects: the dance communication of honeybees (*Apis* spp.). Worker bees ‘waggle dance’ to indicate the distance and direction to key resources such as food, water and potential nest sites (von Frisch, 1967; l'Anson Price & Grüter, 2015). That bees have evolved this capacity suggests that a key constraint on the evolution of such informative signalling is not cognitive capacity, but social structures, such as collective foraging. As Krebs and Dawkins (1984) argued, animals generally have a strong incentive to signal in ways that manipulate others to their own benefit; deception is thus rampant in animal signalling. Among collective foragers, however, group members share a common interest in one another's foraging success. Alexander (1987) noted that, because honeybees feed their sisters, honest communication about food resources promotes their inclusive fitness. Ants are collective foragers as well; they signal food location by depositing a scent trail on their way from good food sources back to the nest (Wilson, 1962). Bees cannot deposit a scent trail through the air when they fly, so they have evolved the waggle dance instead. As collective foraging evolved in hominins, foragers began to share a wider stake in the foraging success of other group members, and so would have had more to gain from honestly indicating the location of good food sources. Bickerton (2009) argues that hominins evolved language to communicate about the location of large animal carcasses, which they scavenged. Insofar as individuals shared food with one another, collective foraging would promote honest communication about other food sources as well. Cooking probably played an important role in language evolution as well, by providing energy needed to grow and maintain larger brains, as part of an autocatalytic cycle, whereby tool use increased the importance of social learning, resulting in greater access to energy to fuel bigger brains, which in turn provided more computational power for insights and social learning.

Living apes provide little evidence that language-like capacities existed in the LCA_PH_. However, insofar as tool use, food processing and collective foraging evolved in hominins because hominins shared with their ape ancestors a requirement for high-quality food, inefficient quadrupedal locomotion and a capacity for tool use, our ape ancestry may have played an important role in leading us to this otherwise improbable path.

## Conclusions

Long-term field studies of chimpanzees at Gombe and other sites have amply fulfilled the hopes of visionaries such as Leakey and Imanishi. Decades of systematic study have revealed both striking similarities between chimpanzees and humans and profound differences. Gaining further insights into the details of these differences, and how and why they are likely to have emerged, has taken decades of work, and much remains to be done.

Contrary to Fuentes (2018), the relevance of chimpanzee studies for understanding human evolution does not depend upon a belief that phylogenetic inertia dooms us to behaving in the ways the LCA_PH_ behaved millions of years ago. We know phylogenetic inertia has limited power from the many ways in which chimpanzees, bonobos and humans differ from one another: each of these living species is the product of continuing evolution. Instead, studying chimpanzees and other primates provides insights into both general factors that favour the evolution of particular traits (such as aggressive vs. friendly behaviour), and into how being a particular kind of animal – a great ape – results in particular constraints and opportunities.

As primates, we have adaptations for complex social living. Yet this alone cannot explain the peculiar trajectory of hominin evolution. Multiple lineages of primates adapted to the expanding open habitats of the Plio-Pleistocene: savanna monkeys, patas monkeys, gelada monkeys, baboons and hominins. Of these, only hominins evolved bipedal locomotion, extensive tool use and language. Monkeys are efficient quadrupeds, and so did not need to evolve bipedal locomotion. Monkeys can digest a broader range of foods, and so did not need to invent tools to obtain high-quality foods – and because they remained quadrupedal, did not evolve bipedal locomotion that freed hominin hands for more effective tool use. The reasons for how and why language evolved remain obscure, but this most distinctive of human traits is probably closely related to the consequences of increasingly elaborate tool use, including an increased importance of social learning, increased access to energy to fuel larger brains and the increased value of honest signalling among collective foragers. Thus, all of these specifically human traits depend on our having evolved from apes. The reason hominins, but not baboons or elephants or some other lineage, ended up on this peculiar path very much depends on our heritage as apes: inefficient quadrupeds with grasping hands, orthograde posture, large brains and digestive systems that require high-quality foods. Long-term studies of apes at Gombe and other sites have been, and will continue to be, essential for providing insights into our own distinctive evolutionary pathway.

## Research transparency and reproducibility

This review paper did not report new findings, apart from the data used to make Figure 4, which are available upon reasonable request of the author.
